# An-Gong-Niu-Huang-Wan (AGNHW) regulates cerebral blood flow by improving hypoperfusion, cerebrovascular reactivity and microcirculation disturbances after stroke

**DOI:** 10.1186/s13020-024-00945-7

**Published:** 2024-05-22

**Authors:** Xiao Zhang, Jiamin Pei, Luping Xue, Zhe Zhao, Renhao Xu, Cong Zhang, Cong Zhang, Lijie Fu, Xiangjian Zhang, Lili Cui

**Affiliations:** 1 Department of Neurology, The Second Hospital of Hebei Medical University, Shijiazhuang, 050000 Hebei China; 2 Hebei Key Laboratory of Vascular Homeostasis and Hebei Collaborative Innovation Center for Cardio-cerebrovascular Disease, Shijiazhuang, 050000 China; 3 School of Electrical Engineering, Yanshan University, Qinhuangdao, 066004 China; 4 Department of Medical Service, The Second Hospital of Hebei Medical University, Shijiazhuang, 050000 Hebei China; 5 Beijing Ruiweisi Pharmaceutical Technology Co., Ltd, Beijing, 100000 China

**Keywords:** An-Gong-Niu-Huang-Wan, Compound Chinese medicine, Ischaemic stroke, Cerebral blood flow, Cerebrovascular reactivity, Microcirculation disturbance

## Abstract

**Background:**

The restoration of cerebrovascular regulation and improvement of cerebral blood flow in ischaemic regions are crucial for improving the clinical prognosis after stroke. An-Gong-Niu-Huang-Wan (AGNHW) is a famous traditional compound Chinese medicine that has been used for over 220 years to treat acute ischaemic stroke; however, its role in the regulation of cerebral blood flow is still unclear. The aim of the present study was to investigate the regulatory effect of AGNHW on cerebral blood flow and microcirculation after ischaemic stroke and to elucidate the underlying mechanisms involved.

**Methods:**

Male C57BL/6 mice were subjected to distal middle cerebral artery occlusion (dMCAO) and randomly assigned to the sham, MCAO, or AGNHW groups. AGNHW was administered intragastrically 1 h after dMCAO. The rotarod test was utilized to evaluate behavioural function; TTC was used to determine the infarct volume; and ischaemic injury was assessed by detecting brain levels of SOD, MDA and NO. Then, cortical perfusion and acetazolamide-induced cerebrovascular reactivity were assessed using laser speckle contrast imaging, and the velocity and flux of red blood cells in cortical capillaries were detected using two-photon laser scanning microscopy. In addition, we employed RNA-Seq to identify variations in gene expression profiles and assessed endothelium-dependent changes in microcirculatory dysfunction by measuring vasoactive mediator levels.

**Results:**

AGNHW significantly increased cerebral blood flow, reduced the infarct volume, and promoted functional recovery after cerebral ischaemia. AGNHW increased the velocity and flux of red blood cells in capillaries and improved cerebrovascular reactivity in the ischaemic cortex. Furthermore, AGNHW regulated endothelium-dependent microcirculation, as evidenced by decreases in the expression of endothelins (Edn1, Edn3 and Ednrb) and the ratios of brain and serum TXB2/6-keto-PGF1α and ET-1/CGRP.

**Conclusions:**

AGNHW improved cerebral hypoperfusion, regulated cerebrovascular reactivity and attenuated microcirculatory dysfunction within the ischaemic cortex after stroke. This outstanding effect was achieved by modulating the expression of genes related to vascular endothelial cell function and regulating endothelium-dependent vasoactive mediators.

**Supplementary Information:**

The online version contains supplementary material available at 10.1186/s13020-024-00945-7.

## Introduction

Precise regulation of cerebral blood flow (CBF) is essential for maintaining normal brain function, and its dysfunction underlies many neuropathologies [1]. Ischaemic stroke is a leading cause of death and disability worldwide, particularly in developing countries [2]. After ischaemic stroke, CBF changes dynamically, which in turn affects infarct formation, expansion, repair, and functional recovery. However, ischaemic stroke also leads to cerebrovascular dysregulation, which disrupts the brain’s ability to maintain CBF [3]. A sustained reduction in CBF in the ischaemic region leads to a lack of glucose and oxygen availability, as well as an imbalance between the energy demand and supply in neurons, glia, and endothelial cells [2]. A cascade of molecular events, including excitotoxicity, oxidative stress and mitochondrial dysfunction, triggers secondary brain injury [4]. Moreover, oxygen and nitrogen free radicals derived from the microvascular wall cause microcirculation disturbances and persistent tissue hypoperfusion [5]. Clinical studies have shown that the prognosis of stroke patients is closely related to tissue perfusion [6]. Cerebral vasodilatation and the formation of new collateral circulation are compensatory mechanisms to maintain constant CBF after ischaemia, but they are accompanied by haemodynamic alterations that impair the metabolic regulation of CBF and microcirculation, leading to abnormal cerebrovascular reactivity [7, 8]. Therefore, restoring cerebrovascular regulation and effectively managing CBF in ischaemic regions are essential for improving the clinical prognosis after ischaemic stroke.

Compound Chinese medicine, which refers to a prescription composed of multiple types of Chinese herbal medicine, has been utilized for over 2000 years to treat stroke patients in China and selected Asian countries [9]. An-Gong-Niu-Huang-Wan (AGNHW) is a famous traditional Chinese compound medicine that has been used for over 220 years to treat brain disorders, specifically acute ischaemic stroke [10, 11]. The classic AGNHW formula (Beijing Tong Ren Tang Chinese Medicine Co. Ltd.) consists of Calculus Bovis, a concentrated powder of Cornu Bubali, Moschus, Margarita, Cinnabaris, Realgar, Rhizoma Coptidis, Radix Scutellariae, Gardeniae Fructus, Radix Curcumae and Borneol. A recent study characterized and quantified the chemical constituents of AGNHW using ultrahigh-performance liquid chromatography–tandem mass spectrometry and identified a total of 205 compounds, mainly bile acids, flavonoids, alkaloids and iridoid glycosides. Among them, 68 major constituents were simultaneously detected in different batches of AGNHW samples [12]. In recent years, a number of experimental studies have reported the potential neuroprotective effects of AGNHW on ischaemic injury [13–16]. Treatment of stroke rats with AGNHW inhibited blood brain barrier damage by reducing peroxynitrite and superoxide levels and inhibiting MMP-9 activity after delayed thrombolysis [17]. Moreover, AGNHW ameliorated cerebral ischaemia/perfusion injury by reversing gut microbiota dysbiosis through the modulation of bacterial abundance [18]. The results of clinical meta-analyses also showed that AGNHW-assisted therapy improved overall response rates and neurological deficit scores in patients with acute ischaemic stroke [10]. To date, the role of AGNHW in regulating CBF after ischaemic stroke has not been investigated. In this study, we investigated whether AGNHW affects the regulation of CBF by dynamically detecting cortical perfusion, capillary red blood cell (RBC) flow characteristics, and cerebrovascular reactivity in stroke model mice and the underlying mechanisms that contribute to this effect.

## Materials and methods

### Animals

Male C57BL/6 mice (SPF, 8–12 weeks, 23–26 g, RRID: IMSR_CRL:27) were purchased from Vital River Laboratory Animal Technology (Beijing, China). All mice were housed in groups with free access to food and water at a relative humidity of 60 ± 5%, temperature of 22 ± 3 °C, and a 12 h light/dark cycle. After at least 7 days of acclimation, the mice were randomly assigned to different groups according to the experimental design. All animal procedures were performed in strict accordance with the guidelines approved by the Animal Care and Use Committee of the Second Hospital of Hebei Medical University (Licence No. HMUSHC-130318, 2023-AE105). All experiments and analyses in this study were performed using a randomized design with blinding of the allocation to treatment and control groups.

### Model of cerebral infarction

Cerebral infarction was induced by permanent occlusion of the right distal middle cerebral artery (dMCA) and common carotid artery [19]. Briefly, the mice were anaesthetized with an intraperitoneal injection of tribromoethanol (400 mg/kg, Sigma‒Aldrich, Cat#T48402-25G). The right common carotid artery was isolated and ligated. The right distal MCA was exposed and coagulated with a cautery device (Bovie, USA). The body temperature was maintained at 37.5 ± 0.5 °C during surgery. Mice with subarachnoid haemorrhage and MCA recanalization were excluded. In the sham model, the right common carotid artery and MCA were isolated but not ligated or occluded.

### Drug administration and grouping

AGNHW was kindly provided by Beijing Tong Ren Tang Chinese Medicine Co., Ltd. (Z11020193, China) and was dissolved in 0.9% saline. AGNHW was administered intragastrically at 1 h after dMCAO and thereafter once daily for 14 days or until sampling. The mice were randomly assigned to the following groups to select the optimum dosage for preliminary experiments: sham (sham model + an equal volume of 0.9% saline), MCAO (dMCAO + an equal volume of 0.9% saline), low-dose AGNHW group (AGNHW-L, dMCAO + 125 mg/kg/d AGNHW), medium-dose AGNHW group (AGNHW-M, dMCAO + 250 mg/kg/d AGNHW) and high-dose AGNHW group (AGNHW-H, dMCAO + 450 mg/kg/d AGNHW). In subsequent experiments, the mice were assigned to three groups: the sham group, the MCAO group and the AGNHW group (AGNHW 450 mg/kg/d). According to the Meeh–Rubner equation, a mouse dose of 450 mg/kg/d AGNHW corresponds to a human dose of 3 g/60 kg/d, which is the usual dose for treating stroke patients.

### Assessment of CBF using laser speckle contrast imaging (LSCI)

Real-time and dynamic imaging of CBF was performed via LSCI (PeriCam PSI, PERIMED, Stockholm, Sweden), which has high temporal and spatial resolution and causes little damage. Briefly, mice were anaesthetized with 2% isoflurane, fixed, and the skull was exposed to measure the regions of interest ipsilateral and contralateral to the infarct. A monitoring laser with a wavelength of 785 nm and a maximum output power of 70 mW was used for LSCI. CBF was measured before and after dMCAO. Imaging was then performed on Days 1, 3, 7 and 14. The value of CBF was expressed in perfusion units (PUs).

### Measurements of superoxide dismutase (SOD), malondialdehyde (MDA) and nitric oxide (NO) levels

Fresh cerebral cortex from the peri-infarct region was extracted, and tissue homogenates were prepared. The levels of SOD (Solarbio, BC0175), MDA (Solarbio, BC0020), and NO (Solarbio, BC1475) were measured using assay kits according to the manufacturer’s protocol.

### Behavioural function assessment

The rotarod test was used to assess balance and limb coordination as measures of sensorimotor function. The mice were trained to run on the rotating rod for 3 days before the test, and their motor function was tested in the uniform acceleration mode 2 days before and 1 (Days 7, 8, 9), 2 (Days 14, 15, 16), and 4 weeks (Days 28, 29, 30) after ischaemic stroke. The speed was increased from 4 to 40 rpm within 300 s. The time that the mice remained on the rod before falling off (falling latency) was recorded, and those that did not fall off within 5 min were assigned a latency of 300 s. The test was repeated 3 times per test day, and the average of the 3 trials was recorded. The body weight of each mouse was also monitored during the rotarod test.

### Measurement of the infarct volume

The infarct volume was determined by performing 2,3,5-triphenyltetrazolium chloride (TTC) staining on Days 1, 3 and 7 after ischaemic stroke. Mice were sacrificed after anaesthesia, and the intact brains were rapidly removed and snap frozen at − 20 °C for 15–20 min. The frozen brains were coronally sliced into six sections. (1.5 mm thick) sequentially and isometrically, immediately immersed in a 2% TTC solution, incubated at 37 °C for 20 min, and then fixed in a 4% paraformaldehyde solution for 24 h. Normal tissue appeared red, while infarcted areas appeared white. A macro-optical zoom microscope (Axio Zoom. V16, Zeiss, Jena, Germany) was used for image acquisition, and ImageJ software was used for analysis.

### Cranial window surgery for in vivo imaging procedures

A previously described method [20] was used to facilitate stable two-photon imaging of the cortex. Briefly, the mice were administered 2 mg/kg dexamethasone intraperitoneally 2 h before surgery to minimize brain swelling during the procedure. Surgery was performed under isoflurane anaesthesia (4% induction, 1–2% maintenance in 1 L/min O_2_). A portion of the right rounded skull (3.0 mm posterior to bregma, 1.5 mm lateral to midline, and 3 mm in diameter) was removed using a cranial drill, fitted with a glass piece, and attached to the skull using glue and dental adhesive along with the head fixator. Two-photon imaging experiments were performed 4 weeks later.

### In vivo two-photon fluorescence imaging of RBC velocity and flux in capillaries

Tracking the movement of RBCs in capillaries is essential for characterizing blood flow and understanding microcirculatory function [21]. Two-photon laser scanning microscopy (TPLSM) was used to track the movement of RBCs in the capillaries of live mice. Prior to imaging, the mice were briefly anaesthetized with 2% isoflurane in oxygen and fixed on a stent, and then FITC-dextran (150 mg/kg; FD2000S, Sigma‒Aldrich) was injected via the orbital vein to label the capillaries. A TPLSM (LSM 880, Zeiss, Germany) consisting of a scanning lens (20 × aqueous mirror, numerical aperture: 1.0) and a femtosecond laser (MaiTai) was used to capture images. The wavelength of the two-photon excitation light was 900 nm, and the pinhole wavelength was 600 nm. Frame scan mode was used to obtain 3D images of cortical vessels by Z-axis scanning, with an acquisition depth of 100–300 μm (layer 2/3). Then, vessels less than 10 µm in diameter were categorized as capillaries. Line scan acquisition was used to record the flow and velocity of individual RBCs in the target capillaries. A longitudinal section of a capillary measuring between 10 and 40 μm in length was scanned at a frequency of 3440 Hz and a speed of 0.97 μs/pixel along the centreline. The process of line scans yields space–time (Xt) images in which each RBC produces an oblique shadow. MATLAB software was used to detect the linear features in the image and calculate the velocity of the RBCs after Radon transformation of the images [22]. RBC flux was determined by calculating the number of RBCs per unit time and quantified using ImageJ software [23]. The full width at half maximum (FWHM) was used to quantify the diameter. A capillary was repetitively scanned 3 times, and the average value was calculated. Microvessels show spontaneous reperfusion within 3 h after cerebral infarction [20]; therefore, we chose 3 h as the first acquisition time. Subsequent imaging was performed on Days 1, 3, 7, and 14 after stroke.

### Assessment of cerebrovascular reactivity (CVR)

CVR is the ability of the cerebrovasculature to adapt in response to a challenge or manoeuvre and is a sensitive indicator of changes in the cerebral vascular reserve capacity. In this study, we detected the CVR by measuring CBF changes in mice after the intraperitoneal injection of 50 mg/kg acetazolamide (ACZ) (Sigma‒Aldrich, St. Louis, MO). We first detected the CVR in normal mice after the ACZ injection to exclude the effect of ACZ on blood pressure and to select an optimal time window for recording; moreover, we recorded blood pressure by volumetric pressure tracing (CODA^®^ Monitoring, AD Instruments, Australia). CBF was recorded for 1 min prior to the ACZ injection and then 30–40 min after the ACZ injection. ACZ-induced experiments were performed at 0 h and at 1, 3, 7, and 14 days after stroke. ACZ-induced changes in CVR were calculated as follows: (maximal CBF after ACZ injection—CBF before ACZ injection)/CBF before ACZ injection × 100%. Then, the apical ischaemic region on the LSCI images was outlined before and after ACZ administration, and the changes in the area were calculated. ACZ-induced changes in the apical ischaemic area were calculated as follows: (ischaemic area after the ACZ injection—ischaemic area before the ACZ injection)/ischaemic area before the ACZ injection × 100%.

### RNA sequencing (RNA-seq) and analysis

RNA-seq was performed to determine the mRNA expression profiles in the ischaemic cortex. Total RNA was extracted from the peri-infarct region at 5 days postischaemia. The purity, concentration and integrity of each sample were verified. Library construction and Illumina sequencing were performed by Shanghai Life Gene Technology Co. PCR products were purified (AMPure XP system), and library quality was assessed using an Agilent Fragment Analyzer 5400 system. Library preparations were sequenced on the Illumina NovaSeq 6000 platform, generating 150 bp paired-end reads. The reference genome and gene model annotation files were downloaded directly from the Genome website. Pure paired-end reads were aligned to the reference genome using HISAT2 v2.2.1. Reads mapped to each gene were counted using HTSeq v0.13.5. Gene expression levels were quantified using fragments per kilobase million (FPKM) values. A differential expression analysis under both conditions was performed using the DEGseq R package (1.28.0) for samples without biological replicates and the DESeq2 R package (1.26.0) for samples with biological replicates. The criteria for significantly differentially expressed genes (DEGs) were a fold change ≥ 1.5 and P < 0.05. Gene Ontology (GO) enrichment analysis of DEGs was performed using the clusterProfiler R package (v3.12.0).

### Real-time quantitative PCR (qPCR) analysis

Real-time qPCR was utilized to evaluate the expression levels of endothelin 1 (Edn1), endothelin 3 (Edn3) and endothelin receptor type B (Ednrb) messenger RNAs (mRNAs). The peri-infarct cortex tissue was collected at 5 days post ischaemia, and total RNA was isolated using an RNA extraction kit (10296028, Invitrogen, Carlsbad, USA) according to the manufacturer’s instructions. Reverse transcription was performed using the Sure Script^™^ first-strand cDNA synthesis kit (Gene Copoeia, Guang Zhou, China) to synthesize complementary DNA (cDNA). cDNAs were amplified with a real-time PCR system (LightCycler^®^480, Roche, Germany) in the presence of Blaze Taq^™^ SYBR Green qPCR mix fluorescent dye. GAPDH was used as an internal reference gene to normalize the expression of the target genes. The primer sequences are shown in Table 1.

**Table 1:** Primer sequences for qPCR

Gene	Forward primer (5′–3′)	Reverse primer (5′–3′)
Edn1	CCTGGACATCATCTGGGTCA	TCTGTGGCCTTATTGGGAAG
Edn3	GCTGCACGTGCTTCACTTAC	GCTGGGAGCTTTCTGGAACT
EdnrB	CTTGGGGGTATGGGGAGAGA	ACGCATCAGACTGGAGTTGG
GAPDH	GGTGAAGCAGGCATCTGAG	TGCTGTTGAAGTCGCAGGAG

### Detection of vasoactive mediators

We collected serum and brain homogenates from the peri-infarct cortex to measure the levels of vasoactive mediators on Day 5 after ischaemia. The levels of vasoactive mediators, including NO (Solarbio, BC1475), ET-1 (Boster Biological Technology Co., Ltd., EK0953), CGRP, TXB2, 6-keto-PGF1α and cAMP (Elabscience Biotechnology Co., Ltd.; E-EL-M0215c, E-EL-M1144c, E-EL-0054c and E-EL-0056c), were quantified using assay kits according to the manufacturer’s protocols or ELISA kits. Standard working curves were generated based on the concentration range of the detected mediators. The OD values were measured using a Tecan SPARK microplate reader. The concentration of each sample was determined by substituting the OD values into the standard curve.

### Statistical analysis

SPSS version 21.0 software was used to perform the statistical analysis. Two-tailed independent t tests or Wilcoxon paired tests were used for two-group comparisons, and one-way ANOVA or the Kruskal‒Wallis test was used for multiple comparisons. The significance level was set at P < 0.05. The data are presented as the means ± SEMs. Each experiment was repeated independently with similar results, and the results were quantified in the corresponding graphs. All the chart graphs were generated using GraphPad Prism 8.0 software. Charts of enrichment analyses were generated using the free online platform for data analysis and visualization, http://www.bioinformatics.com.cn.

## Results

### AGNHW improved CBF in the ischaemic hemisphere during the acute and recovery periods

**Fig. 1:**
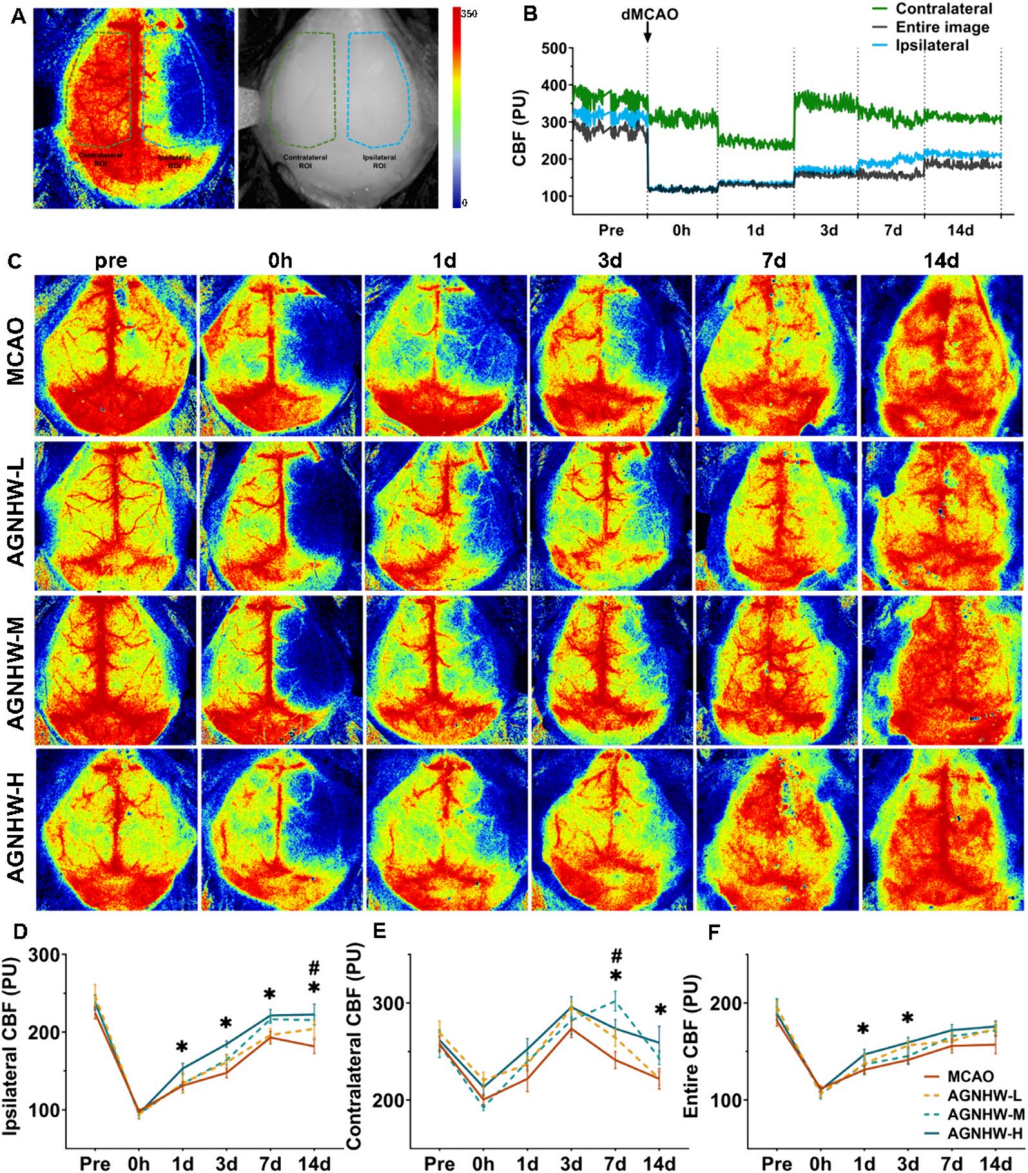
AGNHW improved CBF in the ischaemic hemisphere after stroke. **A** Representative skull and LSCI images displaying the measured areas in the ipsilateral and contralateral hemispheres. The blue dashed line outlines the ipsilateral region, while the green dashed line outlines the contralateral region. **B** CBF changes in ipsilateral, contralateral and whole-brain hemispheres of ischaemic mice at different time points after stroke. Representative LSCI images (**C**) and quantification of CBF (**D**–**F**) in the MCAO, AGNHW-L, AGNHW-M and AGNHW-H groups before and at 0 h, 1 d, 3 d, 7 d and 14 d postischemia. The AGNHW-H group showed a significant increase in ipsilateral CBF at 1 d, 3 d, 7 d and 14 d after ischaemia. The AGNHW-M group showed an increase in the ipsilateral CBF on Day 14 and in the contralateral CBF on Day 7 (n = 12). *P < 0.05 (AGNHW-H group vs. MCAO group); ^#^*P* < 0.05 (AGNHW-M group vs. MCAO group)

The dynamic changes in CBF in the ipsilateral and contralateral hemispheres to the infarct and whole brain (apical region) were measured (Fig. 1A). No significant differences were observed in either the ipsilateral or contralateral CBF among the groups prior to or immediately after ischaemia (P > 0.05). However, the CBF decreased significantly in both the ipsilateral and contralateral hemispheres within 1 day after ischaemia, and the contralateral CBF gradually recovered after 3 days (Fig. 1B). A high dose of AGNHW significantly increased the ipsilateral CBF on Days 1, 3, 7, and 14 (Fig. 1C, D) and increased the whole-brain CBF on Days 1 and 3, indicating an improvement in both the acute and recovery phases after ischaemic stroke. On Day 7 and 14, the AGNHW-H group also exhibited an increase in the contralateral CBF (Fig. 1C, E). The ipsilateral CBF in the AGNHW-M group increased significantly on Day 14 but not at other time points (Fig. 1C, D), whereas the contralateral CBF in the AGNHW-M group increased on Day 7 (Fig. 1C, E). However, the low dose of AGNHW had no effect on postischemic CBF. The results suggested that a high dose of AGNHW notably improved cerebral hypoperfusion in the ischaemic hemisphere postischemia. This therapeutic effect was observed immediately after the acute phase, indicating the potential for early intervention.

### AGNHW alleviated cerebral ischaemic injury and promoted functional recovery after ischaemic stroke

**Fig. 2:**
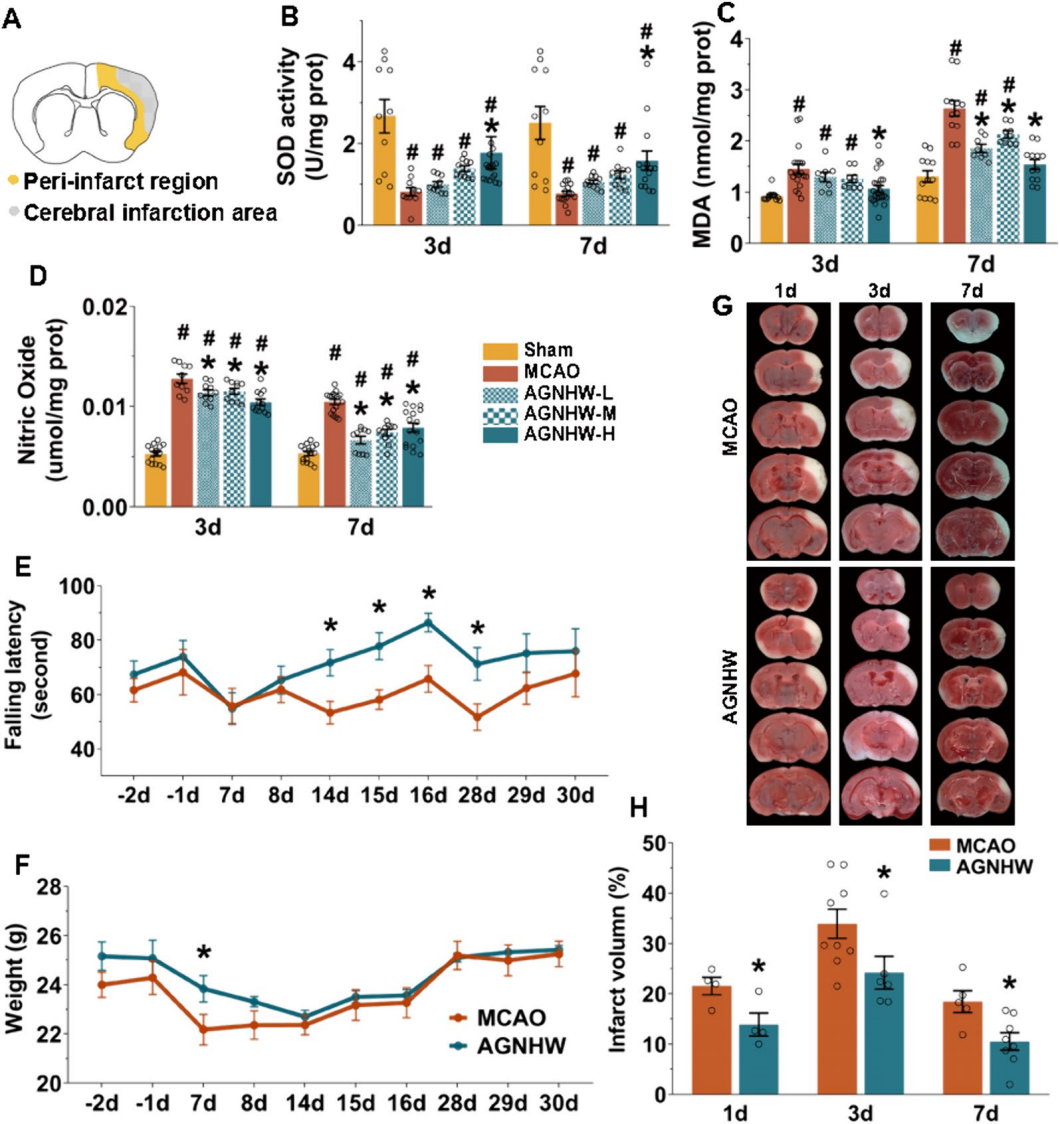
AGNHW reduced ischaemic injury and promoted functional recovery after stroke. **A** Schematic diagram of brain sections showing the infarct and peri-infarct regions. **B**–**D** Quantitative analysis of SOD, MDA and NO levels in the different groups at 3 d and 7 d. A high dose of AGNHW had a significant protective effect on ischaemic injury. **E** Rotarod test showing improved motor function in the AGNHW group at week 2 (14–16 days) and week 4 (28 days). n = 10 for the MCAO group, n = 11 for the AGNHW group. **F** Body weights of the mice during the rotarod test. **G** Representative TTC images and quantitative analysis (**H**) showing that AGNHW significantly decreased the infarct volume. ^#^*P* < 0.05 vs. the sham group; *P < 0.05 vs. the MCAO group

We tested the effect of different doses of AGNHW on ischaemic injury to verify the optimal dose. The antioxidant activity of the SOD enzyme increased in the AGNHW-H group during both the acute (Day 3) and recovery (Day 7) periods of ischaemic stroke (Fig. 2B). The levels of the lipid peroxidation agent MDA and the inflammatory mediator NO were also decreased in the AGNHW-H group (Fig. 2C, D). The MDA content in the AGNHW-M and AGNHW-L groups decreased on Day 7, and the NO content decreased on Days 3 and 7. However, compared with the MCAO group, no significant differences in the SOD levels were observed in the AGNHW-M and AGNHW-L groups. Therefore, the 450 mg/kg dose of AGNHW was used in subsequent experiments because of its significant therapeutic effects.

The results of the rotarod test showed that no significant difference in the latency to fall between the AGNHW and MCAO groups at week 1 (Days 7, 8; P > 0.05). However, at week 2 (Days 14, 15, 16) and Day 28, the AGNHW-treated mice spent significantly longer amounts of time on the rotarod (Fig. 2E), suggesting that AGNHW improved functional recovery after ischaemia. The mice in the AGNHW group gained weight within the first week and at a faster rate than those in the MCAO group (Fig. 2F). Additionally, AGNHW significantly reduced the infarct volume on Days 1, 3, and 7 (Fig. 2G, H). These findings indicated that the AGNHW had a beneficial effect on cerebral ischaemic injury and promoted functional recovery.

### AGNHW regulated the velocity and flux of RBCs in capillaries after ischaemic stroke

**Fig. 3:**
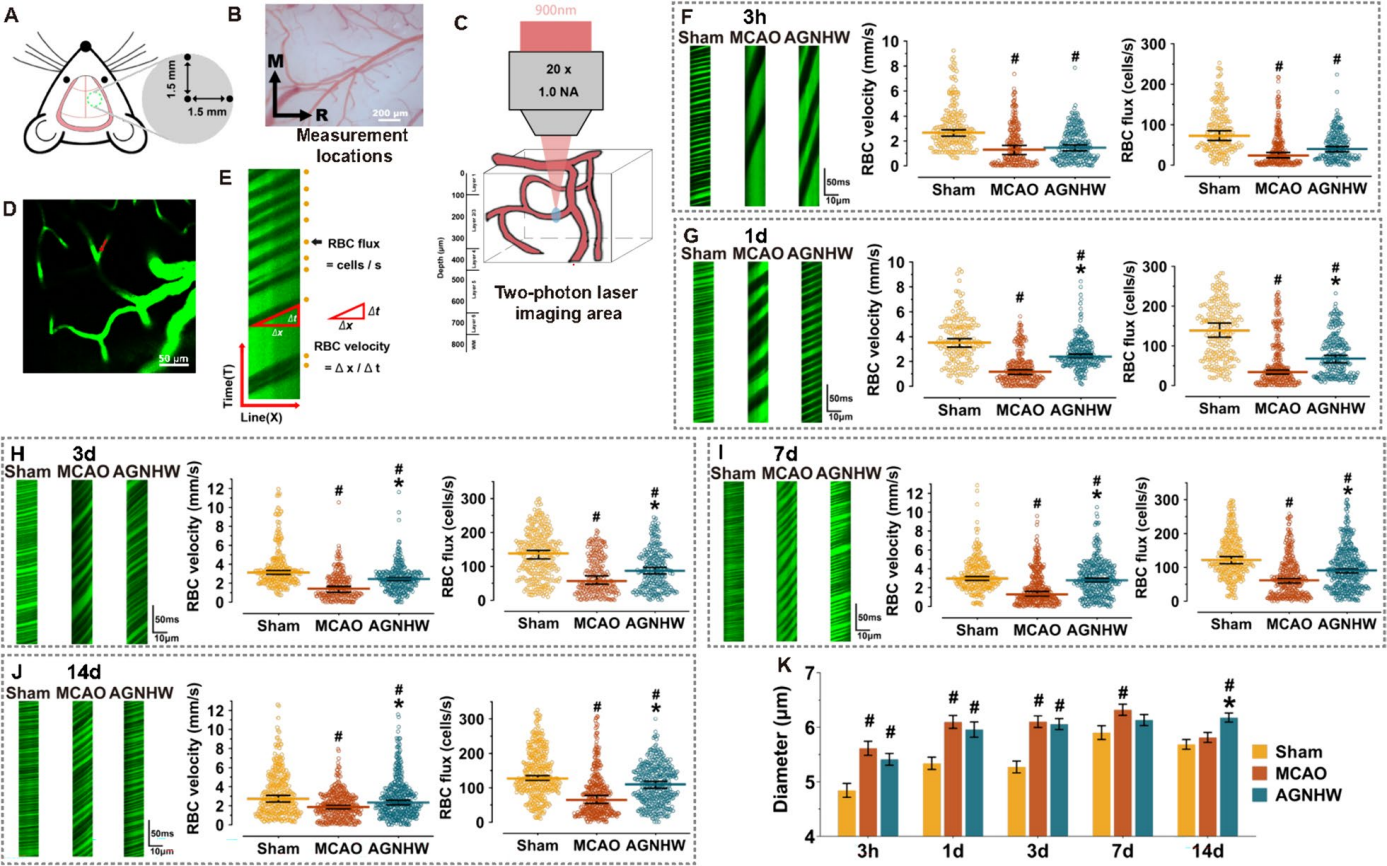
AGNHW regulated the velocity and flux of RBCs in capillaries in the ischaemic region. **A** Schematic diagram showing the scanning location under the cranial window using TPLSM. **B** The scanning area was positioned according to the location of the relevant vessels. R, rostral; M, medial. **C** Schematic diagram displaying the scanning location of capillaries. **D** Maximal projection of a z-stack image of the cortical vasculature. The red arrow indicates the location of the line scan in a capillary. **E** Line-scan image of a capillary for quantifying the velocity and flux of RBCs. The scans were displayed in a space–time image with time increasing from bottom to top. The dark streaks running from the lower left to the upper right were formed by the motion of the nonfluorescent RBCs. The RBC velocity was calculated as the inverse of the slope of the streaks observed. The flux of RBCs was determined by counting the number of streaks. **F**–**J** Representative line-scan images of cortical capillaries and statistical analysis of RBC velocity and flux in different groups after ischaemia. Cerebral infarction resulted in a significant decrease in the velocity and flux of RBCs during the acute (3 h, 1 d and 3 d) and recovery phases (7 d and 14 d). Treatment with AGNHW markedly increased the velocity and flux of RBCs on Days 1, 3, 7, and 14 after ischaemia. **K** Statistical analysis of the increase in capillary diameter in AGNHW-treated mice on Day 14; n = 4. #P < 0.05 vs. the sham group; *P < 0.05 vs. the MCAO group

As a method to investigate the effect of AGNHW on microcirculation, we utilized TPLSM to track the movement of RBCs in capillaries (Fig. 3A–E). TPLSM provides high spatial and temporal resolution, but the signal-to-noise ratio decreases as the imaging depth increases. The use of femtosecond lasers does not improve image quality beyond depths of 300–500 μm. Therefore, we focused on the cortical region between 100 and 300 μm in depth, corresponding to layers 2/3 (Fig. 3C). We found that the velocity of RBCs in capillaries was inversely proportional to the angle of the RBC streak in the image (Fig. 3E). A slow RBC velocity in capillaries was associated with high oxygen extraction, while a low flux indicated low tissue oxygen pressure. At 3 h and 1, 3, 7, and 14 days postischemia, the velocity and flux of RBCs were significantly lower in the MCAO group than in the sham group (Fig. 3F, J), whereas the capillary diameter increased (Fig. 3K), indicating a significant microcirculatory disturbance in the ischaemic cortex after stroke. AGNHW did not affect the velocity or flux of RBCs in the ischaemic region at 3 h (Fig. 3F). On Days 1, 3, 7, and 14, both the RBC velocity and flux were significantly increased in AGNHW-treated mice (Fig. 3G–J). In addition, AGNHW increased the capillary diameter on Day 14 after ischaemia (Fig. 3K). The results suggested that AGNHW ameliorated the microcirculatory disturbance in the ischaemic region by modulating the flux and velocity of RBCs during the acute and recovery phases after ischaemia.

### AGNHW improved the CVR in the ischaemic hemisphere during the recovery period

The effect of AGNHW on the CVR after infarction was evaluated by monitoring alterations in the CBF induced by ACZ injection. First, the optimal time window for ACZ-induced CVR without affecting blood pressure was identified (Supplementary Fig. 1A). After ACZ administration, the CBF of both hemispheres increased progressively, peaking at 30–40 min with a 30% increase and then returning to baseline within 2 h (Supplementary Fig. 1B). Blood pressure was restored to preinjection levels 30–40 min after ACZ administration (Supplementary Fig. 1C). This result indicated that the ACZ-induced increase in CBF at this interval was not influenced by blood pressure.

**Fig. 4:**
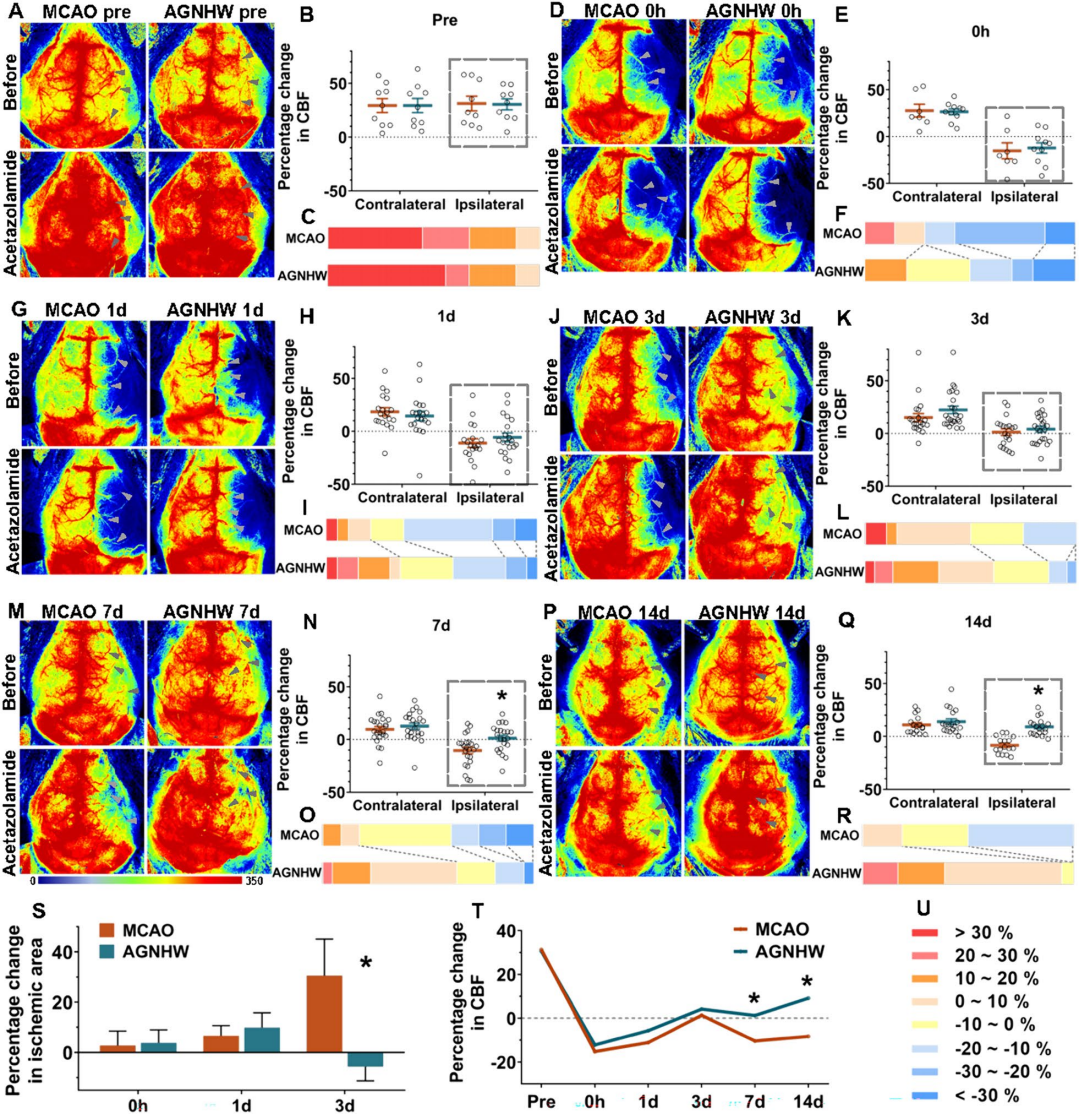
AGNHW improved the CVR in the ischaemic region. Representative LCSI images showing changes in CBF (ΔCBF) before and after ACZ administration in the MCAO and AGNHW groups before (**A**) and at 0 h (**D**), 1 d (**G**), 3 d (**J**), 7 d (**M**), and 14 d (**P**) after ischaemia. The grey arrowheads indicate where changes occurred after the ACZ injection. Statistical analysis of the ΔCBF in the ipsilateral and contralateral regions before (**B**) and at 0 h (**E**), 1 d (**H**), 3 d (**K**), 7 d (**N**), and 14 d (**Q**) after ischaemia. The graph of the ΔCBF composition in the ipsilateral region showing the ratio of positive and negative increases in the ΔCBF in the MCAO and AGNHW groups before (**C**) and at 0 h (**F**), 1 d (**I**), 3 d (**L**), 7 d (**O**), and 14 d (**R**) after ischaemia. AGNHW significantly improved the CVR and increased the ratio of positive ΔCBF in the ischaemic region on Days 7 and 14. **S** AGNHW inhibited the apical ischaemic area (%) enlargement induced by ACZ on Day 3. **T** Line chart showing that the CVR changes over time after ischaemia in the AGNHW and MCAO groups. *P < 0.05 vs. the MCAO group. **U** Changes in CBF (ΔCBF) were categorized into 8 levels from − 30 to + 30%.

Changes in CBF (ΔCBF) were categorized into 8 levels from − 30 to + 30% (Fig. 4). Prior to dMCAO surgery, ACZ induced approximately 26.45 and 28.63% increases in CBF in the ipsilateral and contralateral hemispheres, respectively, with no significant variation between hemispheres (Fig. 4A–C). However, the CVR in the ischaemic hemisphere was significantly impaired after ischaemia. During the two-week period following ischaemia, most animals (particularly stroke model mice in the acute phase) displayed a negative increase in CBF (-ΔCBF) (Fig. 4E, H, K, N, Q) and an enlarged apical ischaemic area (Fig. 4D, G, J, S) after the ACZ injection, suggesting a blood-stealing phenomenon. In the acute phase, AGNHW did not significantly improve the CVR within 3 days (Fig. 4D, E, G, H, J, K), but it increased the ratio of positive ΔCBF in the ipsilateral region on Days 1 and 3 (Fig. 4I, L) and inhibited the ACZ-induced enlargement of the apical ischaemic area on Day 3 (Fig. 4S). The graph of the ΔCBF composition shows the ratio of the inducible haemodynamic response within 2 weeks (Fig. 4F, I, L, O, R). During the recovery period, the CVR in the AGNHW group exhibited a substantial increase on both Days 7 and 14 (Fig. 4M, N, P, Q), with a significant decrease in the ratio of negative ΔCBF (Fig. 4O, R, T). Although the CVR on the contralateral side showed a slight reduction postischemia, no significant difference was observed (Fig. 4D, E, G, H, J, K, M, N, P, Q). The CVR in the MCAO group showed a two-step decrease (Fig. 4T), with the first step occurring immediately after ischaemia (Fig. 4E, F), followed by a gradual recovery in half of the mice on Day 3 (Fig. 4K, L), but the CVR decreased again 1 week later (Days 7 and 14) with the formation of collateral circulation (Fig. 4N, O, Q, R). Three days following ischaemia, the CVR in the AGNHW group began to progressively recover within 2 weeks (Fig. 4T). This result suggested that AGNHW predominantly improved the CVR in the ischaemic hemisphere during the recovery period after ischaemia.

### AGNHW regulated the expression of genes associated with cerebrovascular function

**Fig. 5:**
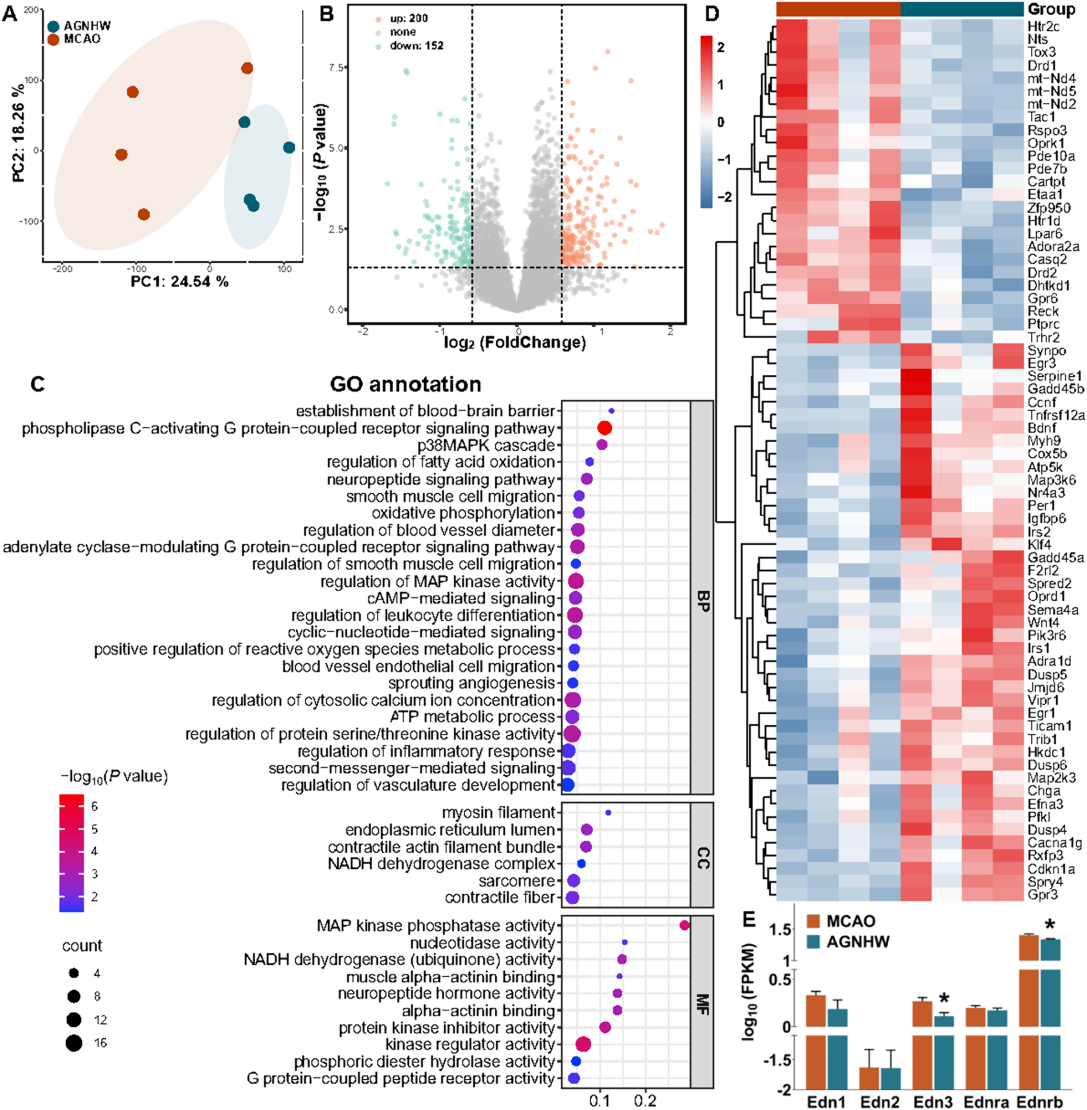
AGNHW regulated the expression of genes associated with cerebrovascular function. **A** PCA revealed distinct clustering of genes in the MCAO and AGNHW groups. **B** Volcano plot showing DEGs between the MCAO and AGNHW groups (fold change ≥ 1.5 and P value < 0.05). **C** GO analysis revealed that the DEGs were associated with the regulation of the blood‒brain barrier and blood vessel diameter. BP, Biological processes; CC, cellular component; MF, molecular function. **D** Heatmaps displaying the expression of DEGs (fold change ≥ 1.5 and P value < 0.05) associated with the regulation of vascular function in the MCAO and AGNHW groups. **E** The statistical analysis of vasoactive mediators showed that AGNHW downregulated the expression of the Edn3 and Ednrb genes. n = 4. *P < 0.05 vs. the MCAO group

RNA-Seq was subsequently conducted to identify DEGs in the ischaemic cortex and to investigate the molecular pathways involved in AGNHW-mediated regulation of microcirculation. Principal component analysis (PCA) revealed distinct clustering between groups, with the first two components accounting for 24.54 and 18.26% of the variance, respectively (Fig. 5A). A total of 352 DEGs were identified (fold change ≥ 1.5 and P < 0.05) in the AGNHW group compared to the MCAO group, including 200 upregulated DEGs and 152 downregulated DEGs (Fig. 5B). The analysis of biological processes revealed that the DEGs were primarily involved in regulating the blood‒brain barrier, blood vessel diameter, MAP kinase activity, oxidative phosphorylation, inflammatory response, cAMP-mediated signalling and G protein-coupled signalling activity. The analysis of cellular component and molecular function terms revealed that these DEGs were significantly associated with contractile fibres, the endoplasmic reticulum lumen, the NADH dehydrogenase complex, MAP kinase phosphatase and NADH dehydrogenase (Fig. 5C). Among these DEGs, 91 were related to the regulation of the cerebral vasculature (Fig. 5D). To identify vasoactive mediator genes associated with the regulation of vasomotoricity, we performed an analysis of all DEGs with a significance level of *P* < 0.05, regardless of fold change, between MCAO and AGNHW groups. This allowed us to capture a wide range of potential candidates that may play a role in mediating vascular function after ischemic stroke. An analysis of all DEGs (P < 0.05) revealed that the expression of genes encoding proteins such as Edn3 and Ednrb was significantly downregulated in the AGNHW group (Fig. 5E). These findings indicated that the AGNHW modulated cerebral haemodynamics by regulating the expression of genes associated with cerebrovascular function.

### AGNHW regulated vasoactive mediators in the cerebral ischaemic region

**Fig. 6:**
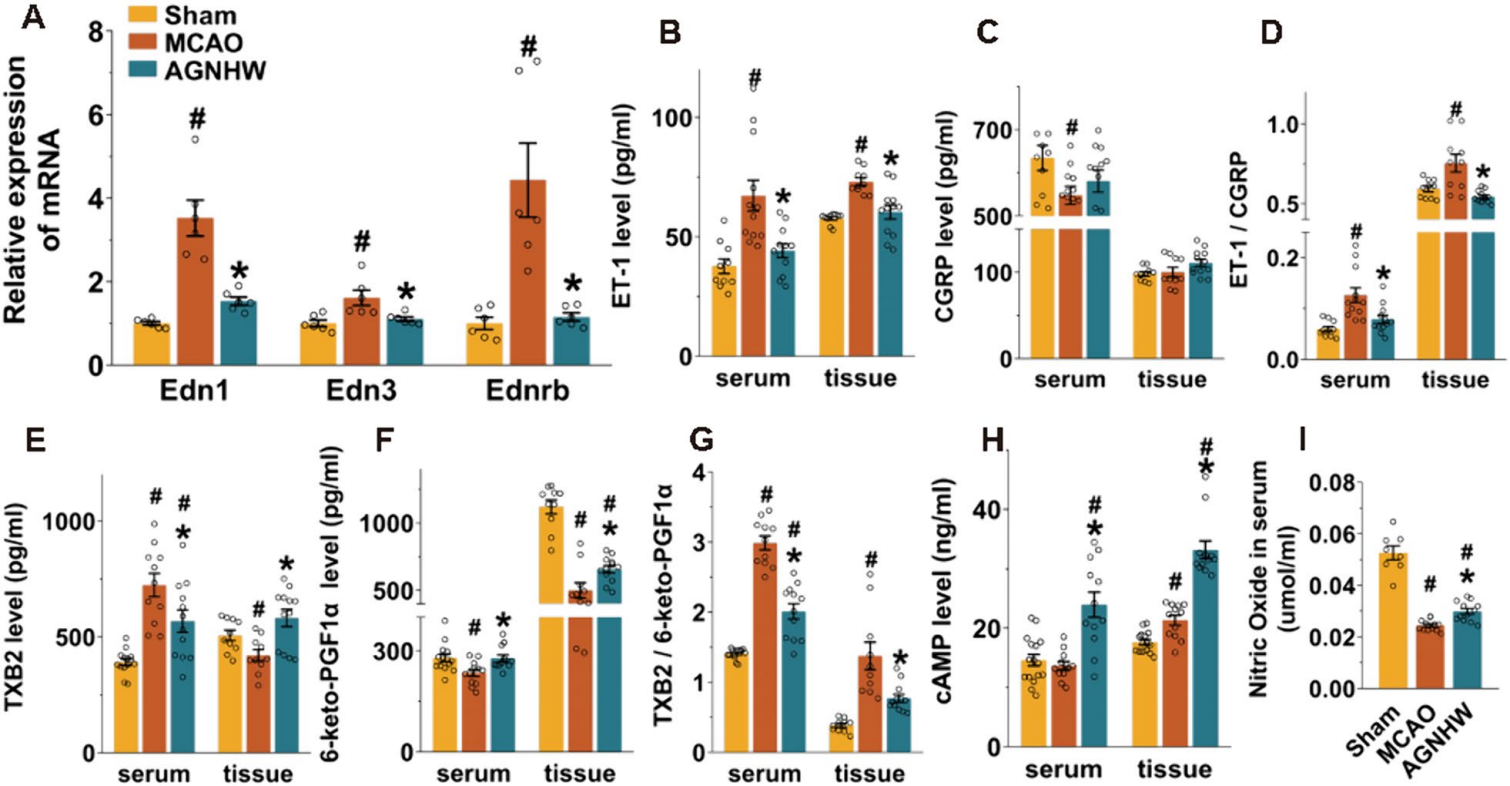
AGNHW regulated vasoactive mediators after ischaemic stroke. **A** AGNHW downregulated the expression of the Edn1, Edn3 and Ednrb mRNAs in the ischaemic cortex on Day 5. **B**–**I** The levels of vasoactive mediators in the serum and brain were examined. AGNHW increased 6-keto-PGF1α, NO, and cAMP levels and decreased ET-1 and TXB2 levels in the serum and brain after ischaemia. The ratios of ET-1/CGRP and TXB2/6-keto-PGF1α in the serum and brain were also increased. ^#^*P* < 0.05 vs. the sham group; *P < 0.05 vs. the MCAO group

The mRNA expression of endothelins in the peri-infarct cortex was then verified by qPCR. We found that AGNHW significantly downregulated the expression of the Edn1, Edn3 and Ednrb mRNAs (Fig. 6A). ET-1, CGRP, TXB2 and 6-keto-PGF1α (stable metabolites of TXA2 and PGI2), NO, and cAMP have been reported to be important vasoactive mediators that regulate vasomotor tone and microvascular function. Ischaemia resulted in significantly lower levels of CGRP, 6-keto-PGF1α, and NO (Fig. 6C, F, I) and higher levels of ET-1 and TXB2 in serum and/or brain tissue (Fig. 6B, E). The ET-1/CGRP and TXB2/6-keto-PGF1α ratios in both the serum and brain were also significantly elevated after ischaemia (Fig. 6D, G). AGNHW significantly increased the levels of 6-keto-PGF1α, cAMP, and NO (Fig. 6F, H, I) and decreased the levels of ET-1 and TXB2 in both the serum and brain (Fig. 6B, E). In addition, the ratios of ET-1/CGRP and TXB2/6-keto-PGF1α (Fig. 6D, G) in the serum and brain were also decreased by AGNHW treatment. These results suggested that AGNHW improved microcirculatory function by regulating vasoactive mediators after ischaemic stroke.

## Discussion

Brain function is critically dependent on the close coordination of metabolic demands, the proper delivery of oxygen and nutrients, and the removal of cellular waste, which requires continuous regulation by CBF [24]. Administering AGNHW for 2 weeks after ischaemic stroke facilitated the long-term restoration of neuronal function, as determined by quantifying motor coordination. In this study, we showed that AGNHW played a beneficial role in regulating CBF after ischaemic stroke, ultimately ameliorating cerebral ischaemia injury. This outcome was achieved by improving three important mechanisms: (1) the cerebrovascular response to hypoperfusion, (2) vascular reactivity to vasoactive stimuli in the ischaemic region, and (3) endothelium-dependent microcirculation disturbances.

The local blood supply plays a crucial role in the short- and long-term recovery of dendritic structures and neuronal function following ischaemic stroke. After a stroke, the cerebrovascular system acts as a regulator, quickly adjusting haemodynamics to accommodate and compensate for injury in the ischaemic region. However, when cerebral ischaemia occurs, these regulatory mechanisms can become dysfunctional, leading to an inability to maintain adequate CBF. This disruption exacerbates the severity of ischaemic injury and amplifies tissue damage [25, 26]. Oxidative damage in the ischaemic region is the result of altered cerebrovascular homeostasis and antioxidant dysregulation. This damage often presents as increased levels of reactive oxygen species and lipid peroxides, leading to elevated superoxide anion levels in the affected tissues. After ischaemic stroke, the overproduction of NO in the brain parenchyma further contributes to this problem by increasing the levels of peroxynitrite (ONOO-) and causing protein nitration. This process, in turn, impairs endothelial cells, increases blood‒brain barrier permeability, and worsens cerebrovascular dysregulation [25, 27]. Using LSCI, our research revealed that the traditional Chinese medicine AGNHW significantly enhanced blood flow in the ischaemic hemisphere following ischaemic stroke (Fig. 1). In addition, AGNHW was found to stabilize cerebrovascular regulation, ultimately leading to a reduction in the infarct size (Fig. 2). These positive effects were attributed to the ability of AGNHW to inhibit oxidative stress-induced injury and the NO-mediated inflammatory response, ultimately facilitating functional recovery poststroke. Our findings highlight the potential of AGNHW as a promising therapeutic intervention for ischaemic stroke.

The efficient delivery of oxygen requires the simultaneous action of at least two regulatory mechanisms: increased CBF and homogenization of microvascular flow [28]. Capillary networks account for the majority of the cerebrovascular length [29]. Traditionally, blood flow is believed to be regulated exclusively by pial arterioles on the brain’s surface and small penetrating arterioles in the parenchyma. These arterioles voluntarily change their diameter after ischaemic stroke to maintain and fully restore flow during reperfusion [30]. However, Rungta et al. revealed that the majority of vascular resistance in the brain is actually located within the capillary network, and the greatest increase in blood flow velocity should occur in the smallest capillaries [31]. Maintaining stable and adequate capillary flow is critical to meet the high metabolic demands of the brain [32]. After ischaemic stroke, an initial decrease in local blood perfusion pressure triggers the self-regulated dilatation of resistance vessels. This process is accompanied by a decrease in the velocity and flux of RBCs [30, 33]. Numerous studies have indicated that a slow RBC velocity is linked to high oxygen uptake, while RBC flux is positively related to the mean oxygen pressure [28, 34, 35]. The reduced RBC velocity and flux after ischaemic stroke indicated disturbances in the microcirculation, which in turn affected the delivery of oxygen and energy to the brain, leading to the exacerbation of tissue damage and enlargement of the infarct. Through TPLSM, we discovered that the diameter of capillaries in the ischaemic cortex increased significantly after ischaemic stroke, while the RBC velocity and flux were much lower than those at baseline, indicating a noteworthy disruption in the microcirculation (Fig. 3). The administration of AGNHW was found to modulate microvascular haemodynamics in both the acute and recovery phases following ischaemic stroke, ensuring an adequate blood supply and oxygen exchange. This effect was achieved by increasing the velocity and flow of RBCs in capillaries, and, consequently, an increase in cortical perfusion was observed in the ischaemic region of AGNHW-treated mice during this period. These findings provide strong evidence that AGNHW plays a positive role in regulating CBF by modulating the cerebrovascular response to hypoperfusion.

The CVR is a crucial indicator of cerebrovascular function. The CVR can be impaired and may even be permanently damaged in cases of severe ischaemic injury, leading to long-term impairments in affected brain tissues. The restoration of energy metabolism may not always be accompanied by an improvement in the CVR [8]. In severe cases, the accumulation of metabolites and tissue acidosis can severely suppress the CVR in the ischaemic core and penumbra, leading to maximal cerebral vasodilation and preventing further responses to hypercapnia [7, 8]. AGNHW did not impact the CVR or the proportion of ACZ-induced blood stealing in the acute phase of ischaemic stroke. However, the enlargement of the ischaemic area caused by blood stealing was significantly reduced after AGNHW treatment, suggesting a better and faster recovery of cerebrovascular function. AGNHW significantly improved the CVR in the ischaemic region after 2 weeks (Fig. 4). This improvement was thought to occur in two ways: (1) the AGNHW attenuated the cumulative production of harmful mediators, and (1) it may have enhanced the CVR in newly formed collateral anastomoses during the recovery period [36].

Endothelial cells play a critical role in regulating vascular tone through a variety of mechanisms, including the activation of electrical signalling, vasodilators, and vasoconstrictor mediators such as NO, bradykinin, prostacyclin and endothelin [27, 37]. These endothelium-dependent reactions play a crucial role in controlling CBF and microcirculatory function [24]. AGNHW-regulated genes play a crucial role in blood brain barrier establishment. They also contribute to the regulation of blood vessel diameter and the modulation of cAMP signalling pathways, which are essential for cerebrovascular function (Fig. 5). The qPCR results also revealed that AGNHW inhibited the expression of the genes encoding endothelin and its receptor (Fig. 5). The intricate involvement of these genes in these processes highlights the significance of AGNHW in cerebrovascular regulation. The delicate equilibrium between the production of vascular PGI2 and the formation of its physiological antagonist TXA2 plays a crucial role in maintaining cardiovascular homeostasis [27, 38]. ET-1 and CGRP are another pair of vasoactive mediators. ET-1 is considered the most potent endogenous vasoconstrictor, and changes in its levels can serve as a marker of endothelial dysfunction [39]. On the other hand, CGRP is a vasodilator that counteracts the contractile effects of ET-1 and helps to stabilize the microcirculation [40]. The stable plasma metabolites of PGI2 (6-keto-PGF1α) and TXA2 (TXB2) and the ratios of TXB2/6-keto-PGF1α and ET-1/CGRP are commonly used to assess microcirculation disturbances after ischaemia [41, 42]. NO plays a complex and multifaceted role in pathophysiology. In neurons, it acts as a mediator of glutamate excitotoxicity and contributes to free radical-induced damage through the formation of peroxynitrite. On the other hand, in endothelial cells, it has the opposite effect, inducing vasodilation, enhancing blood flow and reducing hypoxic injury [33]. AGNHW significantly improved microcirculation disturbances by modulating the expression of endothelial vasoactive mediators after ischaemia, as evidenced by increased serum levels of 6-keto-PGF1α, NO, and cAMP and decreased levels of ET-1 and TXB2. In addition, the ratios of ET-1/CGRP and TXB2/6-keto-PGF1α were relatively balanced by AGNHW (Fig. 6). These findings suggested that AGNHW regulated CBF by effectively improving endothelium-dependent microcirculation and may be a promising treatment for ischaemic stroke.

## Conclusions

Our study showed that the famous compound Chinese medicine AGNHW significantly reduced ischaemic injury and increased CBF in the ischaemic region. These changes led to improved functional recovery after permanent cerebral infarction. The therapeutic effect of AGNHW began in the acute phase and continued into the recovery period after stroke. AGNHW positively regulated CBF after stroke by improving the cerebrovascular response to hypoperfusion, vascular reactivity to vasoactive stimuli, and endothelium-dependent microcirculatory disturbances. These changes, in turn, ameliorated ischaemic injury and promoted functional recovery. Our study suggested that AGNHW may play a promising therapeutic role in the treatment of ischaemic stroke by regulating cerebral perfusion and microcirculation.

### Supplementary Information

Supplementary Material 1: Fig 1. ACZ-induced cerebrovascular responses in normal mice. Representative perfusion images **(A)** of typical CBF responded to ACZ over time in normal mice. Quantification changes of regional CBF **(B)** and blood pressure **(C)** in normal mice after ACZ injection. n= 3.

## Data Availability

Data will be made available on request.
