# Supplementary material for: An-Gong-Niu-Huang-Wan (AGNHW) regulates cerebral blood flow by improving hypoperfusion, cerebrovascular reactivity and microcirculation disturbances after stroke
Source: Chin Med. 2024 May 22;19:73. doi: 10.1186/s13020-024-00945-7 (PMC11112936; doi:10.1186/s13020-024-00945-7)
Supplement: Supplementary file 1 — Supplementary Material 1: Fig 1. ACZ-induced cerebrovascular responses in normal mice. Representative perfusion images (A) of typical CBF responded to ACZ over time in normal mice. Quantification changes of regional CBF (B) and blood pressure (C) in normal mice after ACZ injection. n= 3. [file 13020_2024_945_MOESM1_ESM.pdf]

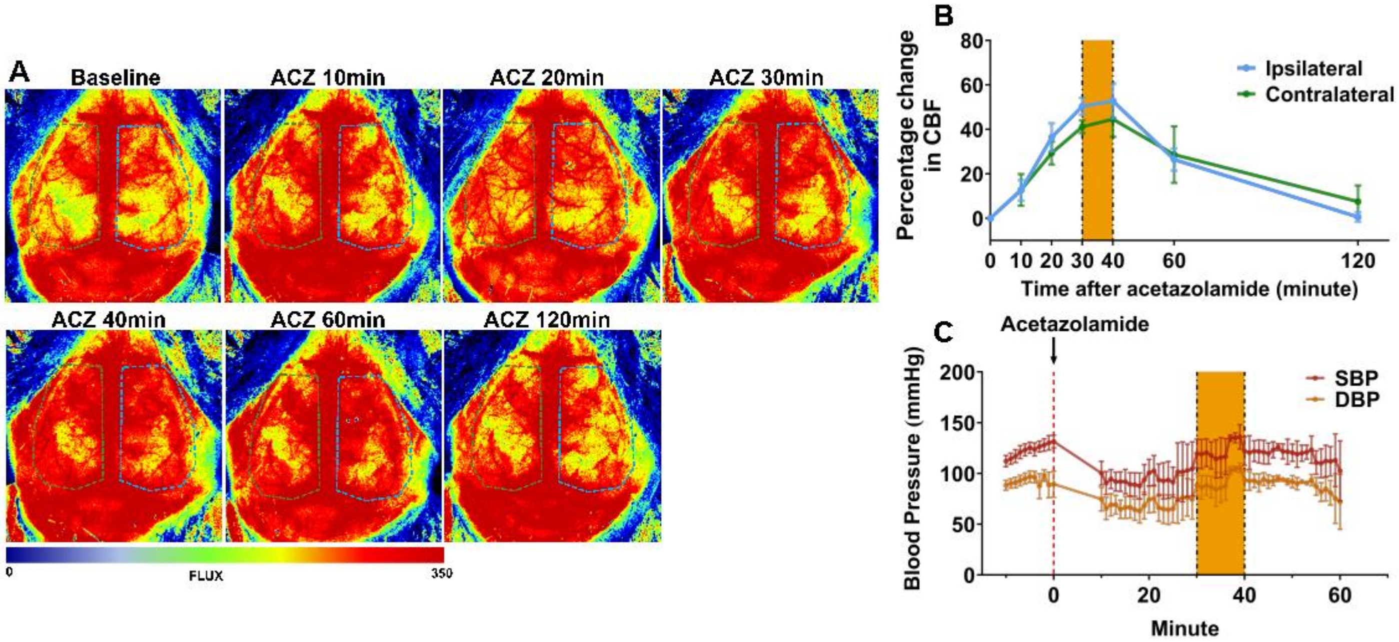

Supplementary Fig 1. ACZ-induced cerebrovascular responses in normal mice. Representative perfusion images **(A)** of typical CBF responded to ACZ over time in normal mice. Quantification changes of regional CBF **(B)** and blood pressure **(C)** in normal mice after ACZ injection. n= 3.
